# Patient-reported impairment following TKA is reduced when a computationally simulated predicted ideal alignment is achieved

**DOI:** 10.1007/s00167-022-07225-7

**Published:** 2022-11-29

**Authors:** Joshua Twiggs, Brad Miles, David Parker, David Liu, Andrew Shimmin, Brett Fritsch, Justin Roe, Jonathan Baré, Michael Solomon, David Dickison, Stephen McMahon, Richard Boyle, Len Walter

**Affiliations:** 1 360MedCare, Suite 3, Building 1/20 Bridge St, Pymble, Sydney, NSW 2073 Australia; 2 Sydney Orthopaedic Research Institute, Sydney, 2067 Australia; 3 Gold Coast Centre for Bone and Joint Surgery, Gold Coast, 4221 Australia; 4 Melbourne Orthopaedic Group, Melbourne, 3181 Australia; 5 North Sydney Orthopaedic and Sports Medicine Centre, The Mater Hospital, Sydney, 2060 Australia; 6 Sydney Orthopaedic Specialists, Sydney, 2031 Australia; 7 Peninsula Orthopaedics, Sydney, 2099 Australia; 8 Malabar Orthopaedic Clinic, Melbourne, 3181 Australia; 9 Boyle Orthopaedics, Sydney, 2042 Australia

**Keywords:** Total knee arthroplasty (TKA), Kinematics, Joint dynamics, Computational simulation, Outcome, PROMS, Machine learning

## Abstract

**Purpose:**

Joint dynamics following Total Knee Arthroplasty (TKA) may influence patient-reported outcome. Simulations allow many knee alignment approaches to a single patient to be considered prior to surgery. The simulated kinematics can be matched to patient-reported outcome to predict kinematic patterns most likely to give the best outcome. This study aims to validate one such previously developed algorithm based on a simulated deep knee bend (the Dynamic Knee Score, DKS).

**Methods:**

1074 TKA patients with pre- and post-operative Computerised Tomography (CT) scans and 12-month post-operative Knee Injury and Osteoarthritis Outcomes (KOOS) Scores were identified from the 360 Med Care Joint Registry. Landmarking and registration of implant position was performed on all CT scans, and each of the achieved TKAs was computationally simulated and received a predictive outcome score from the DKS. In addition, a set of potential alternative surgical plans which might have been followed were simulated. Comparison of patient-reported issues and DKS score was evaluated in a counter-factual study design.

**Results:**

Patient-reported impairment with the knee catching and squatting was shown to be 30% lower (*p* = 0.005) and 22% lower (*p* = 0.026) in patients where the best possible DKS result was the one surgically achieved. Similar findings were found relating attainment of the best tibial slope and posterior femoral resection DKS plans to patient-reported difficulty straightening the knee (40% less likely, *p* < 0.001) and descending stairs (35% less likely, *p* = 0.006).

**Conclusion:**

The DKS has been shown to correlate with presence of patient-reported impairments post-TKA and the resultant algorithm can be applied in a pre-operative planning setting. Outcome optimization in the future may come from patient-specific selection of an alignment strategy and simulations may be a technological enabler of this trend.

**Level of evidence.:**

III (Retrospective Cohort Study).

## Introduction

Following total knee arthroplasty (TKA), there remains a portion of patients who report dissatisfaction or ongoing functional impairment postoperatively (PROs) [4]. One of the major decision points in performing a TKA is the alignment of the components, with the primary concerns being longevity of the implant and patient outcome from the surgery [19].

Studies have shown differences in resultant patient outcomes driven by alignment strategy [7, 11], but other studies have failed to replicate this finding [31, 34]. Similarly, knee joint dynamics (kinematics and kinetics) have been shown to be influenced by alignment strategy, but the relationship is complex and the published literature contradictory [5, 33]. In addition to alignment, patient variable factors such as ligament offsets [22] and bone and gap shape [3, 24] as well as component geometry design [18, 32] will also contribute to the complex dynamic outcome of TKA.

Simulations of knee dynamics are a tool that might be used in understanding this complex outcome. In previous work, the authors have described a computational simulation of patient-specific knee motion undertaking a deep knee bend [35] that has been previously mechanically validated [22]. This simulation has further had a Machine Learning (ML) model of patient outcome generated [23, 26], called the dynamic knee score (DKS). ML models in general are a relatively underexplored [14] tool in TKA, but one with many potentially valuable applications being explored [13]. The DKS has not previously been independently validated in terms of patient outcome prediction. This study’s aim was to validate the DKS in a counter-factual study design that models a surgical decision to either follow the best performing prediction or not. Our hypothesis is the DKS is shown to be a valid tool in pre-operative optimisation of component position and orientation in primary TKA.

## Methods

All primary TKA patients operated on between Jan 2017 and August 2020 with pre- and post-operative CT scans and a 12-month post-operative outcome survey were extracted from the 360 Med Care Joint Registry for inclusion in this study. The registry was established for the purpose of evaluating joint dynamics using a published multi-body dynamics model [2]. Nine experienced surgeons (all with at least 10 years of experience as knee specialist orthopaedic surgeons) performed the surgeries and contributed to the registry, using a variety of surgical techniques including manual instrumentation, printed PSI guides and computer navigation. A total of 1074 patients with complete datasets were extracted from the registry for this study.

### Data collection

12-month post-operative survey data collection within the registry was performed using a previously described web application [25]. Patients were emailed personalized links to a questionnaire to ensure data matching to the correct record. Patients were then free to fill in the questionnaire from their own device of choice. In cases where email contact was not possible, patients filled out the questionnaire over a telephone interview. The survey itself consisted of relevant questions extracted from the KOOS (Knee Injury and Osteoarthritis Outcome Score [21].

Both the pre-and post-operative CT scan imaging was taken using an identical protocol. This protocol requires the patient to lie supine with both legs extended. A full capture of the lower legs with slice thicknesses of a maximum 1.25 mm (machine dependent) in all axes are taken. While there was not a minimum thickness, engineers resample incoming CT scans to 1.25 mm for and higher resolution datasets received to reduce processing time. The protocol is fully described in a prior publication [28]. All radiology centres were trained on the protocol and qualified by engineers under the direction of the lead study author to ensure consistency.

**Fig. 1:**
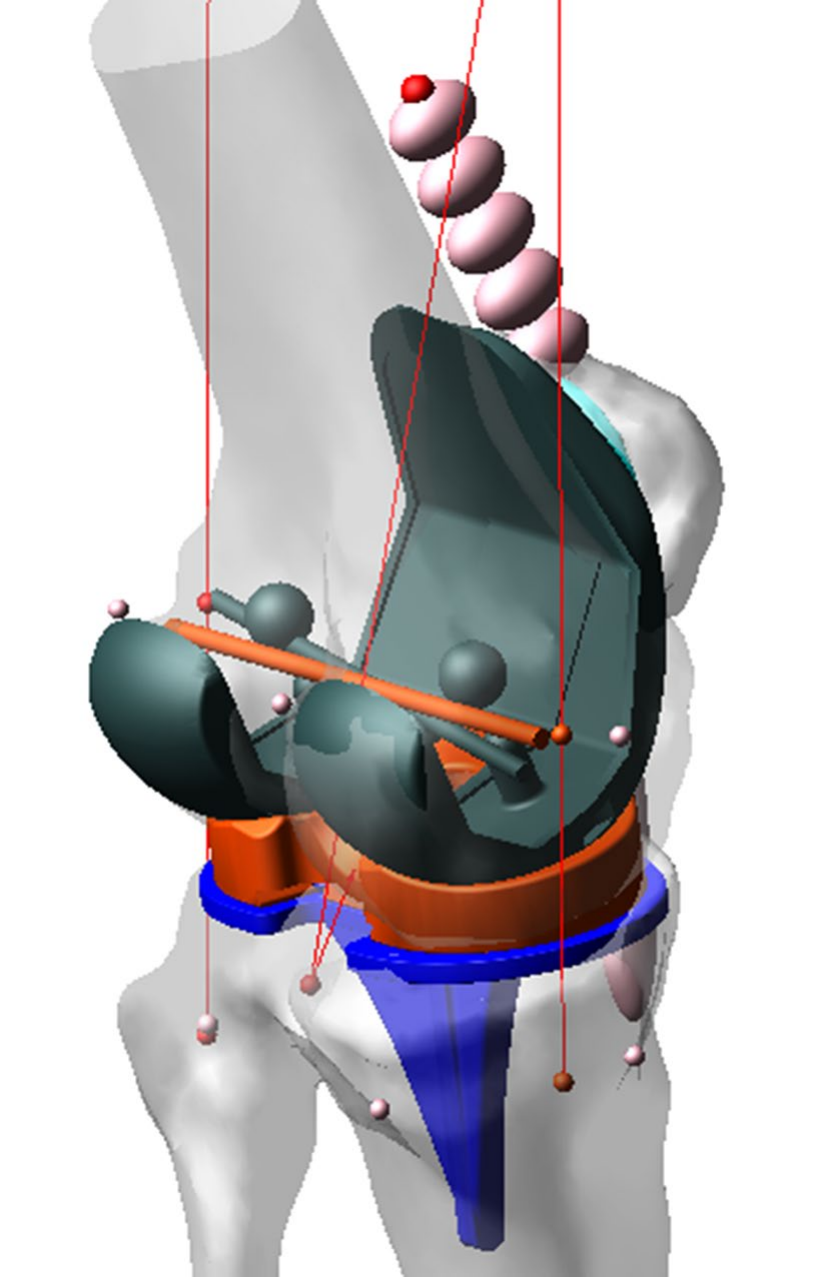
The patient-specific simulation in early flexion during the extension (active) portion of the cycle

3D reconstructed patient femora and tibiae were generated using the imaging software ScanIP (Simpleware, Exeter UK) from pre-operative CT scans [6]. Landmarks describing bone and soft-tissue references are identified by trained engineers qualified by the lead study author used to define patient-specific bone axes and soft-tissue attachment sites [22, 26]. The pre-operative models and landmarks were registered to the post-operative CT scan. In addition, 3D implant models of the implanted components were also registered to the post-operative CT scan. This whole process has been previously shown in a validation study to produce maximum errors of 0.9° ± 0.6° and 0.5 mm ± 0.3 mm with an intra-class correlation coefficient (ICC) > 0.93 indicating excellent reliability [28]. These models, landmarks and component positions were used to produce a computational simulation (see Fig. 1). The simulation replicates a deep knee bend performed in an Oxford Knee Rig (OKR), and includes modelled collateral ligaments, a quadriceps tendon and other passive soft-tissue restraints. All ligaments were modelled as one or two bundles of nonlinear springs as described by Abdul-Rahman et al. [1] with fixed parameters further adapted using a process previously described by Theodore et al. [22, 23, 26]. In this way, the model captures the combination of patient-specific elements, component geometry and component position and orientation that contribute to the dynamic joint motion.

### Model evaluation

**Fig. 2:**
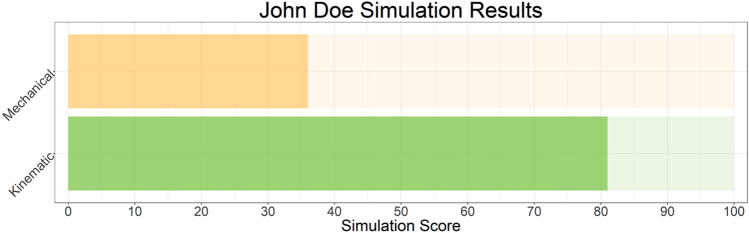
An example of the output of the DKS, an algorithm that reduced the results of the simulation into a rating of the compatibility of those simulated kinematics with a good outcome

DKS model performance was evaluated with a counter-factual study design. First, the achieved surgical result and implant position was simulated. Then, a set of alternate surgical plans the surgeon might have followed were simulated. From each simulation, the DKS score is generated, giving a score from 0 to 100 that predicts the probability of a Patient Acceptable Symptom State (PASS) being reached, using cut off values [12] and via a process described in a previous publication [23]. Patients were grouped by whether (a) the surgically achieved position scored the highest DKS of the variants or (b) whether an alternative simulation scored higher, which would indicate an alternate approach to the surgery was predicted to be more likely to reach a PASS outcome (Fig. 2).

The alternate simulation sets used to cover other surgical plans covered (a) altered posterior resections (+3 mm to – 3 mm resection in 1mm increments), (b) altered tibial slopes (0–12 degrees in 3-degree increments) and (c) altered femoral rotation (matched to the Posterior Condylar Axis (PCA) or matched to the surgical Transepiconylar Axis (TEA)). This gives a total of 13 simulations per patient, which combined with their simulation of actual surgically achieved position amounts to 14 simulations per patient. Evaluation questions were compiled from the KOOS questionnaire to investigate for a relationship to the specific simulated kinematic response expected from the investigated component alignment change. For slope and posterior resection, the following three questions relating to Range of Motion (ROM) were evaluated: “Can you straighten your knee fully?”, “Can you bend your knee fully?”, “Do you experience difficulty descending stairs?”. For femoral rotation, responses to three questions focused on patellar function were evaluated: “Does your knee catch or hang up when moving?”, “Do you feel grinding, hear click or any other type of noise when your knee moves?” and “Do you experience difficulty squatting?”.

### Statistical evaluation

Patients were divided into two groups for an analysis–those where the surgically achieved alignment had the highest DKS (DKS recommendation achieved) and those where a higher scoring alternative plan was identified (DKS recommendation not followed). Validity was assessed by comparing the incidence of patient-reported difficulty or pain in cases between these two groups. As such, the study is a virtual analysis of the DKS as a pre-operative predictive tool, with analysis reflecting whether the surgery matched the highest scoring plan from a set of hypothetical alternative options. Incidence rates are reported with sample results in series, in addition to the sample probability and 95% confidence intervals calculated using the Clopper-Pearson method. Descriptive continuous data series were reported as mean ± standard deviation. Chi-squared tests were used to evaluate differences in patient-reported difficulty incidence between the two groups. All analysis was performed in R 3.6.1.

### Ethics

Ethics approval for the registry and this study was approved by Bellberry Human Research Ethics Committee (Sydney, Australia, approval 2012-03-710).

## Results

A total of 1074 patients were found in the registry and included in the study (586/54% female) with a pre-operative and post-operative CT scan and a completed post-operative survey. Age across the population was 69.6 ± 9.4; mean female age was 70.3 ± 8.7 and mean male age 69.3 ± 10.5, which was significantly different (*p* < 0.001).

**Fig. 3:**
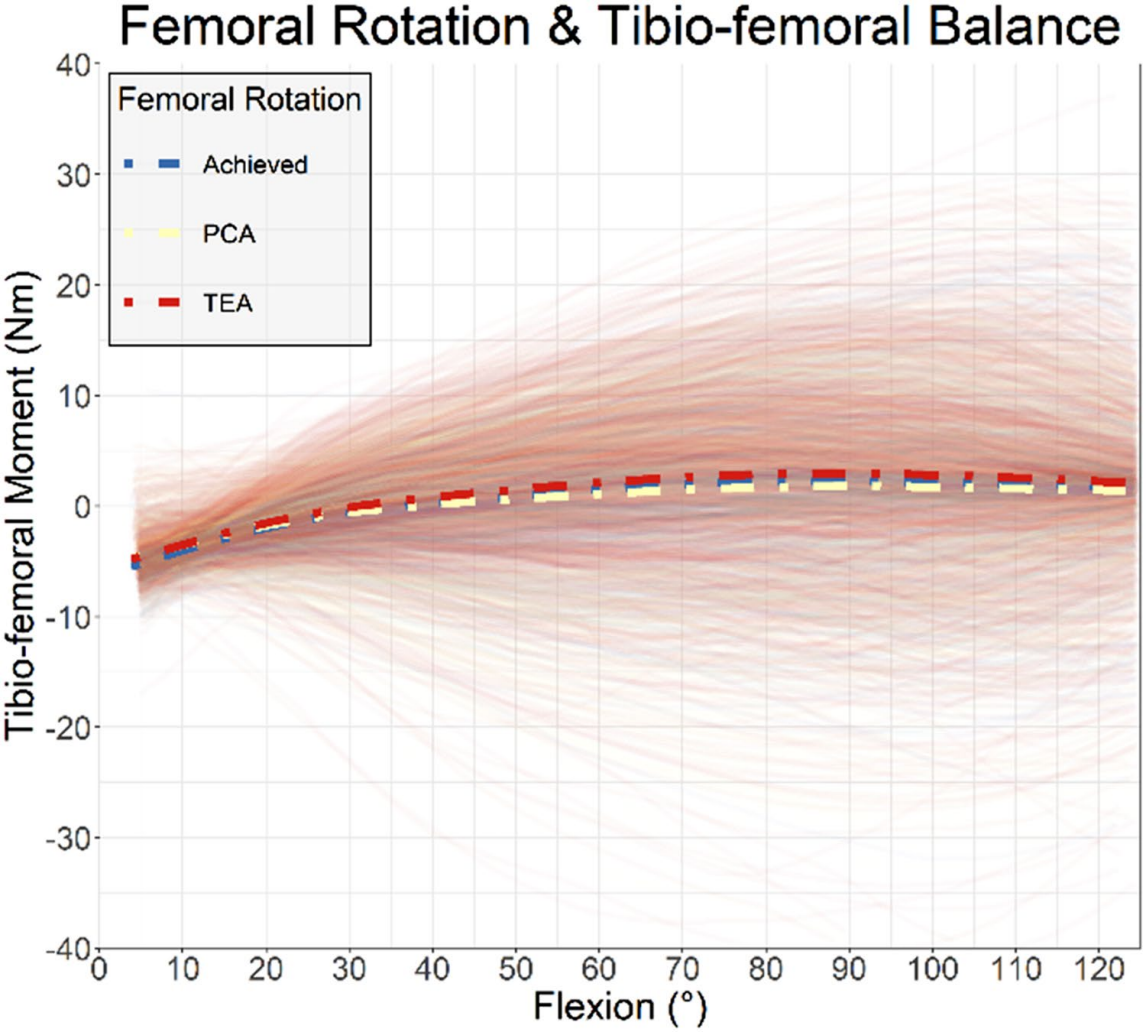
Tibio-femoral coronal plane moment by rotation (either PCA/TEA or that surgically achieved)

In the DKS recommendation achieved group for femoral rotation, 80 of 437 patient-reported difficulty with their knee catching (18.3%, CI [37.7–43.7]), while 163 of 637 patients when the DKS was not followed reported difficulty (25.6%, CI [22.2–29.2]), a 30% (*p* = 0.005) reduction. Similarly, in the DKS recommendation achieved group 97 of 437 patients reported difficulty squatting (22.2%, CI [18.4–26.4]), compared to 180 of 637 (28.3%, CI [24.8–31.9]) when the DKS was not achieved, a 22% (*p*=0.026) reduction. From the kinematic results of the DKS simulation, femoral component rotation altered the medio-lateral tibial-femoral balance but the effect was smaller than that attributable to patient variation across the population (Fig. 3).

**Fig. 4:**
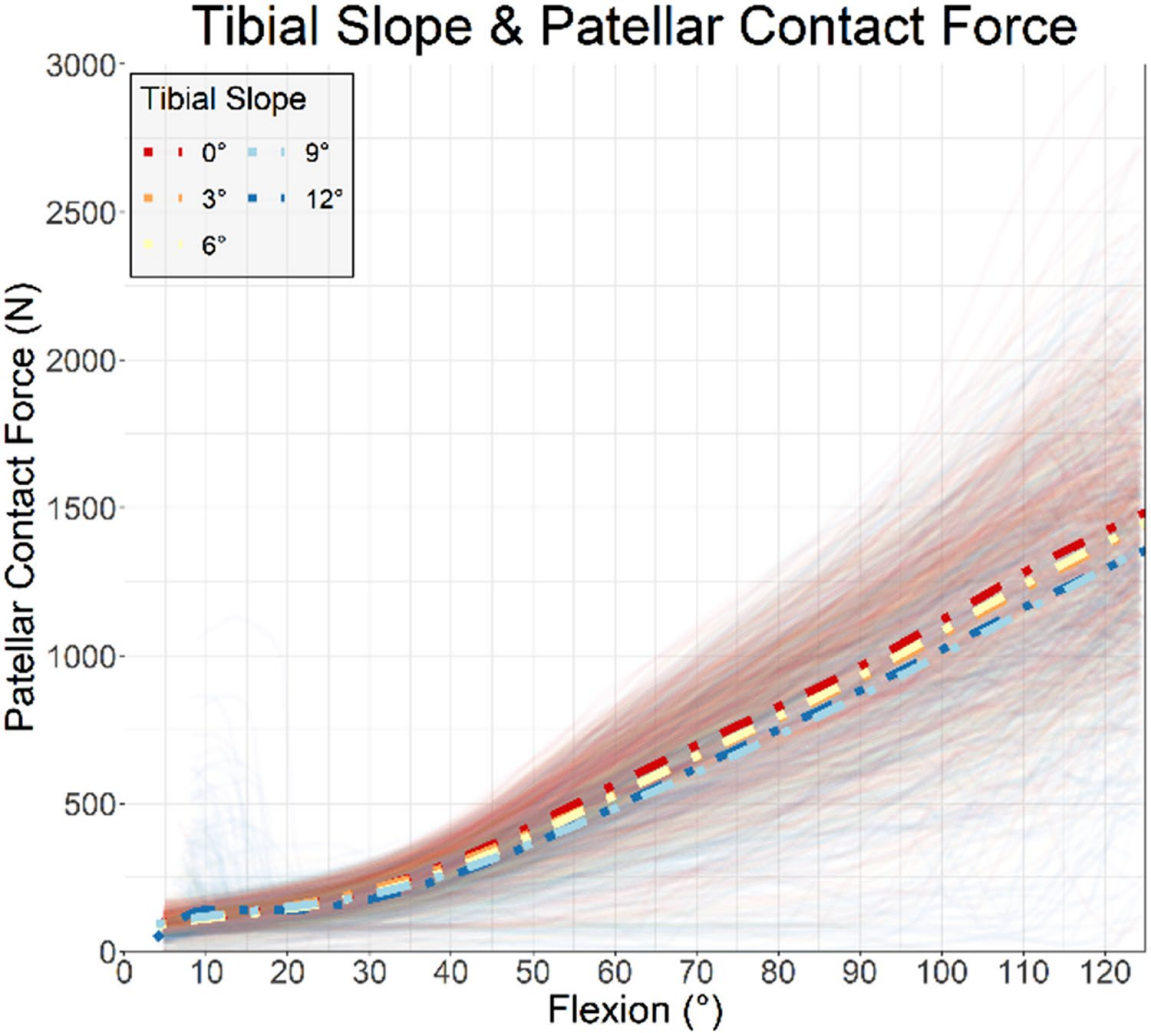
Tibial slope and simulated patellar force

In the DKS recommendation achieved group for tibial slope, 61 of 516 patients reported difficulty straightening the knee (11.8%, CI [9.2–14.9]) compared to 111 of 558 when the best DKS was not followed (19.9%, CI [16.7–23.5]), a 40% (*p* < 0.001) reduction. Likewise, 57 of 516 best DKS recommendation patients report difficulty descending stairs (11.0%, CI [8.5–14.1]) compared to 94 of 558 in the DKS not followed group (16.8%, CI [13.8–20.2]), a 35% reduction in (*p* = 0.006). Kinematic results from the DKS simulation showed the lower tibial slopes of 0°, 3° and 6° degrees had a distinctly higher patellar contact force in flexion (Fig. 4).

**Fig. 5:**
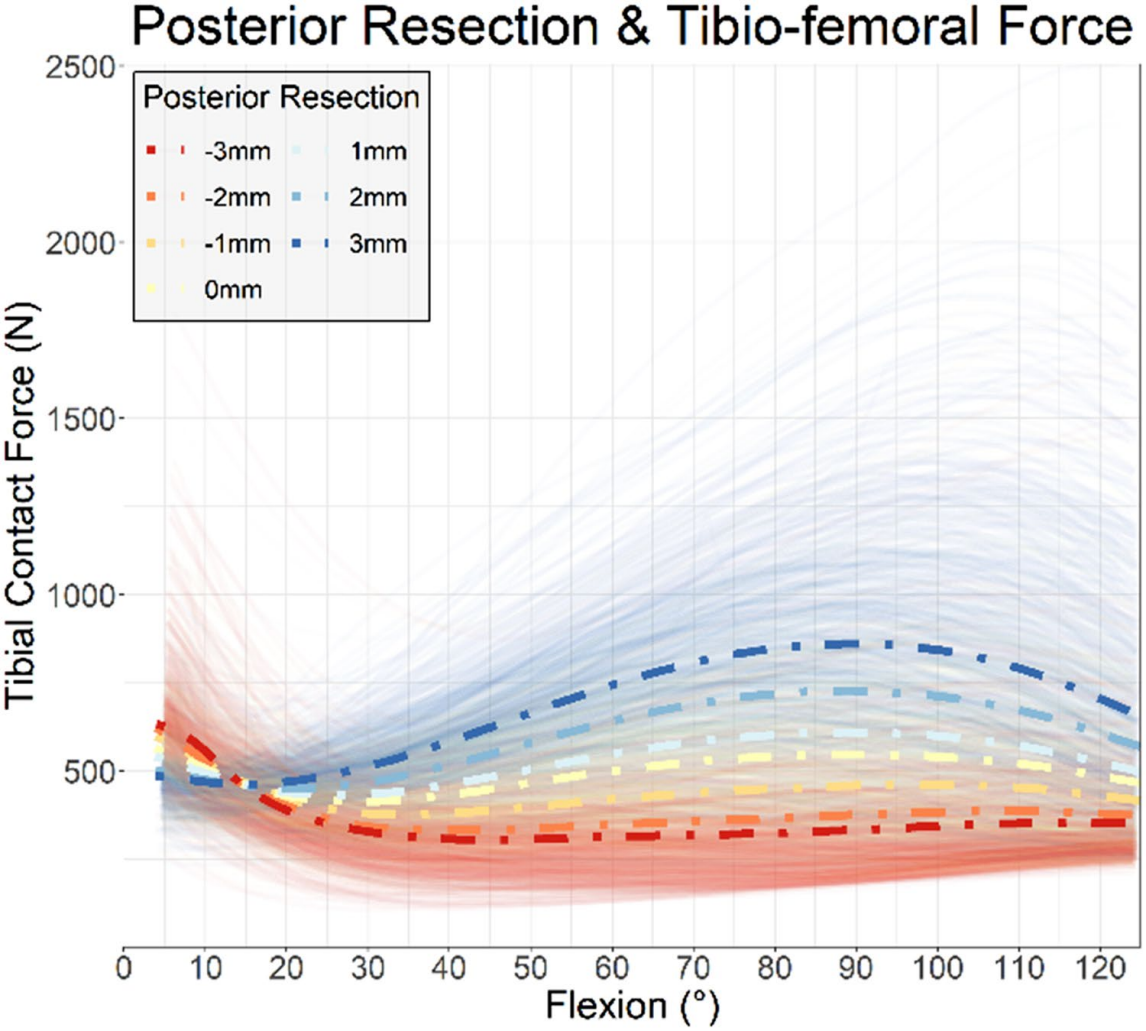
Posterior resection simulated and variation in tibio-femoral contact force through the flexion cycle

In the DKS recommendation achieved group for posterior resection, 64 of 572 patients reported difficulty straightening their knee (11.1%, CI [8.7–14.1]) in the DKS recommended group, while 163 of 637 patients when the DKS recommendation was not achieved reported difficulty (21.5%, CI [18.0–25.4]), a 48% (*p* = 0.001) reduction. The DKS simulation had large differentiation in tibio-femoral contact force simulation results in flexion based on posterior resection (Fig. 5).

A further supplementary analysis was performed focussed on the group of patients reporting difficulty straightening the knee and the DKS recommendations for slope and posterior resection. 169 (15.7%, CI [13.6–18.1]) of patients surgically achieved both the slope and posterior resection DKS recommendation, 446 (41.5%, CI [38.6–44.5]) achieved 1 and 459 (42.7%, CI [39.8–45.8]) had neither. When the achieved position was the highest scoring DKS in terms of both slope simulations and posterior resection simulations, 5 of 169 patients reported difficulty straightening their knee (3.0%, CI [1.0–6.8]), compared to 77/446 (17.3%, CI [13.9–21.1]) and 81/459 (17.6%, CI [14.3–21.4]) when the achieved surgical position was the highest scoring DKS in one or neither of the sets of simulations (*p* < 0.001).

## Discussion

The most important finding of the study is that when the surgical position achieved most closely follows that predicted to give the highest probability of a PASS, instance of a number of specific patients reported functional difficulties was reduced. This provides validation for the DKS as a predictive tool of outcome. The first finding of this study indicated reductions in knee catching and difficulty squatting when the position achieved was the highest DKS result of the femoral component rotation simulations. Femoral component rotation has been show to relate to anterior knee pain [2], can be expected to impact the flexion space and flexion medio-lateral balance [16], the recreation of the trochlea and patello-femoral kinematics [17], which vary by patient [20]. A plot of the coronal plane moment (Fig. 3) showed significant variation in valgus or varus moments (lateral or medial tightness), but population means were similar (indicating trends were primarily patient specific, which might be expected given the variation in rotational axes between patients[24]).

Reduced incidence of patient difficulty straightening the knee was found when the achieved tibial slope or posterior resection matched the highest scoring DKS. Imbalanced flexion gaps have been previously shown to have numerous further impacts on the kinematic outcome of the operation [30], and increased tibial slope has similar previously identified relationships [15, 29]. The findings relating to slope and posterior resection were further shown to compound on each. If following the plan with the highest DKS score is to be considered a recommendation, then when no, one or both recommendations were followed then the position of patients reporting difficulty went from 18 to 16% to 3%. It is important to note that this analysis was supplementary in nature, however, and this finding should be interpreted with caution.

As the optimal targets for alignment in TKA remain unclear, it is possible a single alignment philosophy may not be optimal in all patients [17]. Techniques such as gap balancing have been used to optimize specific component placement decisions (such as femoral rotation and posterior resection) for some time independent of a specific alignment philosophy [10]. New surgical technology is enabling more systematic position optimizations in pursuit of balance, including robotic gap balancing [27] and pressure sensor capability [9] and leading to the concept of Functional Alignment (FA) [8]. Computer simulations with scoring algorithms are potentially powerful adjunct tools to these technologies. A virtual trialling of the final dynamic result of a TKA means many more combinations of component positions could be investigated than is practical in physical surgical trialling. As the trialling is virtual, this could be done without the need for recuts if an alternate position is needed. At its simplest, such tools might be used for selection of an initial alignment prior to the operation. This study demonstrates potential utility in such an approach and is to the author’s understanding the only study to date to validate such a tool relative to patient outcomes.

There are several limitations to this study. One limitation is the fixed nature of the ligament properties used within the model in modelling the collateral ligaments, patella and quadriceps tendons. Furthermore, sample size, while over 1000 patients, would ideally be larger. This study showed an interaction between one of seven alternate plans for posterior resection and five for tibial slope, and the impact of considering multiple interacting effects is multiplicative of the sample requirement. Similarly, the study would benefit from a stricter study design to improve the level of evidence it provides, ideally involving randomization of use of the DKS pre-operatively. These limitations are inherently at odds, as achieving an RCT design with over 1000 patients is a difficult undertaking to resource but would produce a stronger study and data set for this validation work. Further validation in a prospective study context is called for.

Further, within this study, specific functional patient questions have acted as a surrogate for measuring specific functional responses. A better understanding of the performance of the DKS developed might be obtainable with actual functional measurements from patients such as range of motion assessments. The results of these comparisons to selected questions included several findings of no significance, suggesting limits on the degree to the efficacy of the DKS algorithm. It is not entirely clear the degree to which these limits reflect the measurement methodology or the DKS itself, and comparison to actual functional assessments is called for. Additionally, nine surgeons contributed patients into this study. The intent here was to find generalizable rather than tightly controlled findings, but the included surgeons may not be representative of a general orthopaedic surgeon population.

## Conclusion

An algorithmic score determined from joint dynamic measurements from a computational model of a deep knee bend (the DKS) has been shown to correlate with presence of patient-reported impairments post-TKA across a sample of 1074 patients. The resultant algorithm can be applied in a pre-operative planning setting and outcome optimization in the future may come from patient-specific selection of an alignment strategy.
